# Chronic destructive arthritis as an isolated symptom of familial Mediterranean fever (FMF) in a 17 year old Turkish boy

**DOI:** 10.1186/1546-0096-9-S1-P231

**Published:** 2011-09-14

**Authors:** G Dueckers, K Siepermann, G Ganser, S Hardt, M Wennmacher, AE Horwitz, C Haneke, T Niehues

**Affiliations:** 1 HELIOS Children’s Hospital, Krefeld, Germany; 2 St. Josef Stift, Sendenhorst, Germany; 3 HELIOS Clinic for Orthopaedic, Trauma and Hand Surgery; 4 HELIOS Department for Radiology

## Background

FMF belongs to the group of autosomal-recessive inherited fever syndromes. Recurrent fever episodes are typically associated with polyserositis. One complication of this fever syndrome is amyloidosis, thus early colchizine treatment is indicated in FMF.

## Methods

For 11 years a Turkish boy (HLA B 27 negative) suffers from chronic athralgia predominantly of the left hip. No history of trauma, no signs of fever, no abdominal pain and no other physical complaints. Repeated MRI of symptomatic region was classified as multifocal non bacterial osteitis (NBO) of greater trochanter, Os pubis, Os ileum and Ilsosacral joints. Despite the long-term use of NSAID, MTX and systemic and intraarticular Glucocorticoids, no enduring therapeutic response was achievable. Athralgia progressed and range of movement in the left hip decreased dramatically within the last months.

## Results

**Figure 1:**
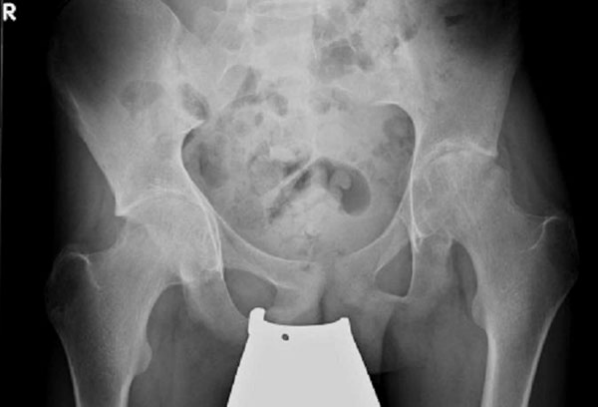
x-ray of pelvis

**Figure 2:**
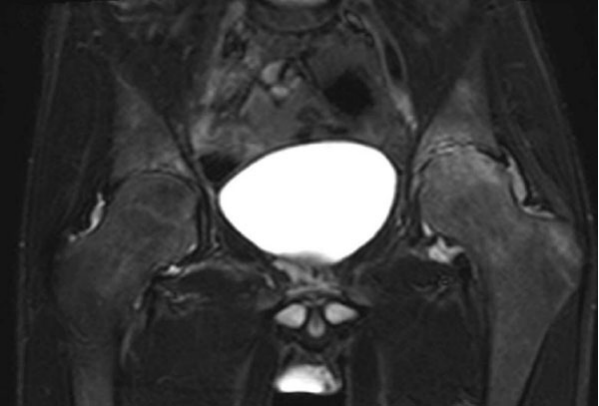
MRI (STIR) of pelvis

Recent diagnostic imaging shows destructive arthritis of left hip (Figure 1 and 2). Before escalation of therapy, the boy has been genetically diagnosed for FMF (homozygous mutation, Exon 10 *MEFV*, Metionin-694 (ATG)>Val (GTG)-/pMet 694VAL-/M 694V). Therapy with colchizine was initiated after diagnosis and relieved the patient’s complaints immediately. Total hip replacement will be performed for the left hip after end of pubertal growth.

## Conclusion

Clinicians should be aware of FMF can present solely as arthritis, without fever and without recurrent episodes of abdominal pain. Chronic destructive Arthritis can be a severe complication with long term sequel.

